# Rainwater chemistry observation in a karst city: variations, influence factors, sources and potential environmental effects

**DOI:** 10.7717/peerj.11167

**Published:** 2021-04-20

**Authors:** Jie Zeng, Guilin Han

**Affiliations:** Institute of Earth Sciences, China University of Geosciences (Beijing), Beijing, China

**Keywords:** Rainwater chemical, Ionic composition, Source identification, Influence factors, Karst urban area

## Abstract

The rainwater chemistry and related air contaminants are used to investigate the rainwater ions sources, variations, and influence factors from 2012 to 2014 in Guiyang city (the typical karst urban area of Southwest China). According to temporal rainwater ion concentrations, the obvious variations were presented in the study period, such as Ca^2+^ (125∼6,652 μeq L^−1^) and SO_4_^2−^ (11∼4,127 μeq L^−1^). Consequently, Ca^2+^, Mg^2+^, SO_4_^2−^ and Cl^−^ are considered as the leading ions. Three critical influencing factors of rainwater ions concentrations, including sources variations, rainfall amount and long-distance migration (rainfall amount > 100 mm) are identified. Based on the typical ionic ratios, source identification suggested that anthropogenic inputs mainly contributed to F^−^, NO_3_^−^ and SO_4_^2−^, while the dusts (crustal sources) are the primary sources of Mg^2+^, Ca^2+^ and K^+^. Cl^−^ Enrichment in long-distance transport is the main contributor of Cl^−^. According to the observation of high level of total wet acid deposition, the more detailed spatio-temporal monitoring of rainfall-related acid deposition (particularly sulfur deposition) is required to understand its potential environmental effects in the aquatic ecosystem of the earth surface.

## Introduction

Wet deposition (rainwater) is a significant sink of atmospheric contaminants, such as sulfur/nitrogen oxides and aerosol (Benedict et al., 2018; Martins et al., 2019; Wei et al., 2019; Wu et al., 2012; Yang et al., 2019). Two processes including in- and below-cloud procedures are the keys to the elimination of atmospheric pollutants during the entire precipitation process (Gonçalves et al., 2002; Xiao et al., 2013), and the chemical components and pH are varied concomitantly (Facchini Cerqueira et al., 2014; Larssen et al., 2006). The chemistry-varied rainwater in different environments can further influence the chemical elemental redistribution and biogeochemical cycles on the earth-surface ecosystem, particularly, aquatic ecosystems (Bhattarai et al., 2019; Martins et al., 2019). Furthermore, exploring the rainwater chemistry is beneficial for estimating the local atmospheric quality, which could be applied to identify the sources of atmospheric pollutants based on various physical-chemical procedures of different pollutants (Cereceda-Balic et al., 2020; Rouvalis et al., 2009; Wei et al., 2020). The previous work has categorized three types of major ions (chemical species) origins in rainwater, including curst sources (terrestrial dust), marine sources (sea-salt input), and anthropogenic sources (human emission) (Jain, Madhavan & Ratnam, 2019; Liu et al., 2013; Xu et al., 2015). All of the source variations, local meteorology, geomorphology, and environmental protect guidelines affect the rainwater chemical species (Cable & Deng, 2018; Szép et al., 2018).

A large number of studies in developed regions (e.g., Europe and the United States) have shown that the rainwater chemical components are relatively homogenous on the large temporal-spatial scale due to the common environmental precautions and unified economic development model (Keresztesi et al., 2019; Keresztesi et al., 2020b; Mullaugh et al., 2015). However, the regional economic development is extremely uneven in rapidly developing China (Wang et al., 2017b). During the different stages of social-economic development, air contaminants types vary greatly (Cable & Deng, 2018; Cong et al., 2015), further changing rainwater chemical species in the environmental system (Jin et al., 2019; Xu et al., 2015). This kind of heterogeneity of rainwater chemistry particularly occurs in the less developed regions of Southwest China (e.g., Guizhou Province), where is the typical continuous distribution area of karst landform over the world (Liu, Han & Zhang, 2020). The rainwater-related studies involved in various karst environmental types (urban, forest and agriculture) (Wu et al., 2012; Xiao et al., 2013). In addition, under the unique geological background of karst landform (such as karst depression and sinkholes), rainfall dominates the migration of surficial material, which makes biogeochemical cycling more sensitive to rainfall event (Qin et al., 2020; Wang et al., 2020; Xu et al., 2020; Yue et al., 2019).

In view of the importance of rainfall processes in karst regions, the in-depth investigation of rainwater chemical compositions in the region is necessary, which significantly benefits the better environmental management and improves the knowledge of karst ecological geochemistry. However, the discussions on acid wet deposition (in the form of rainwater) and its potential environmental effect in karst area in related-studies are relatively rare, so there have been few results for comparison across China.

To advance further knowledge of rainwater chemicals in karst urban area, this study took Guiyang, the most typical karst city in SW China, as a case to conduct a systematic investigation based on the 26 monthly mixed rainwater samples collected in May 2012 to June 2014. The aims are to: (1) investigate the rainwater chemical components in the study period, (2) clarify the seasonal variations of rainwater ions and their influencing factors, (3) identify the potential sources of rainwater ions, and (4) explore the possible impacts of rain-associated acid deposition on environment.

## Materials & methods

### Background of study area and sample collection

**Figure 1:**
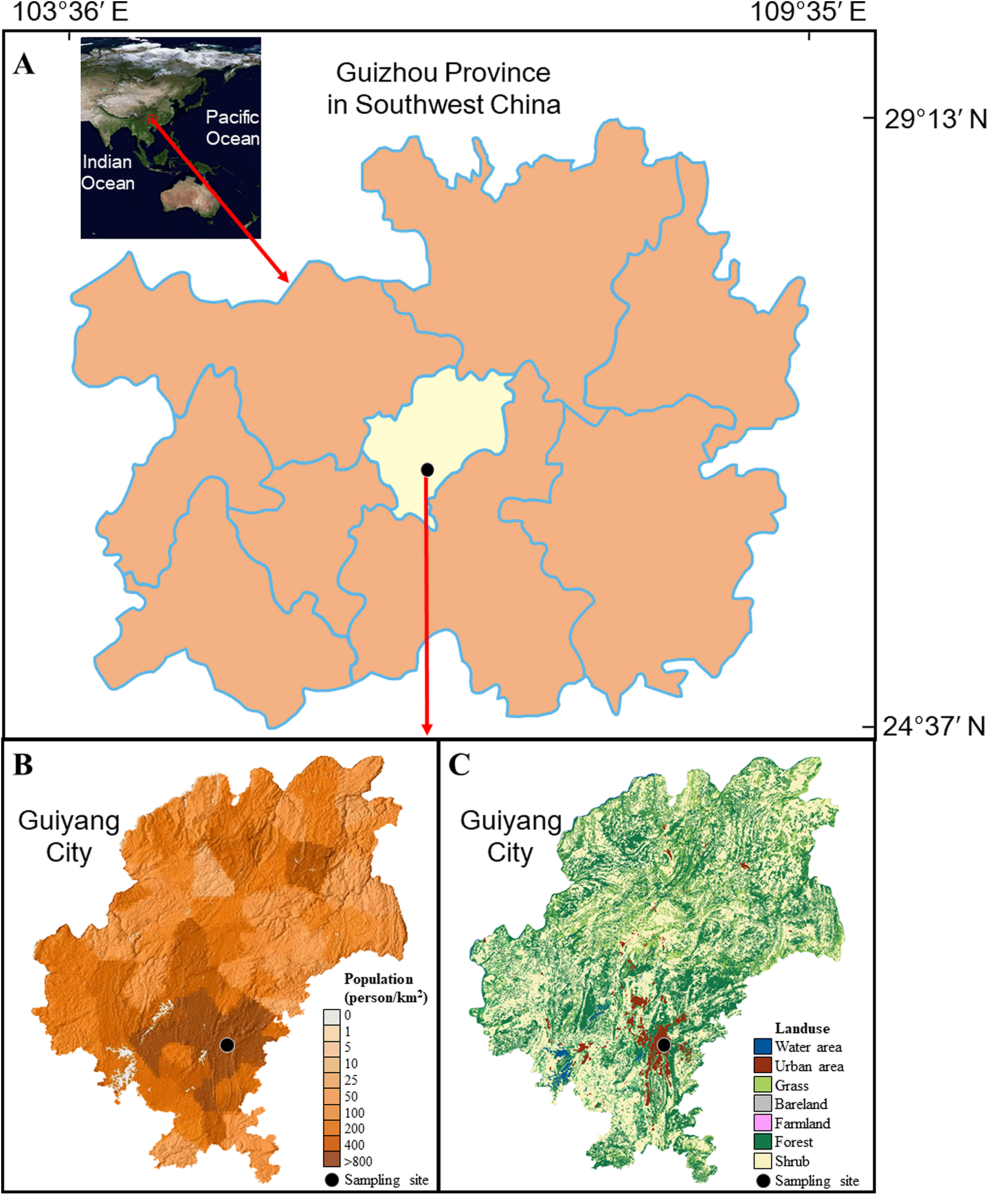
Background of sampling site. (A) the position of Guiyang city; (B) the population density of Guiyang city; (C) the land use of Guiyang city, more details of the surrounding of sampling site can be found in Fig. S1. The data was from Computer Network Information Center of the Chinese Academy of Sciences.

Guiyang City is the capital of Guizhou Province with a landmass of 8,034 km^2^, located in Southwest China (Fig. 1A), which is one of the continuous largest karst regions over world (Zeng & Han, 2020). This city is encircled by hills (elevation ~1,000 m). The karst geomorphology is widespread developed in Guiyang city and carbonate and clastic rocks control the lithology. Subtropical monsoon climate is the characteristic of this city (air temperature ranges from −7.3 to 35.1 °C, with a yearly average of 15.3 °C; average relative humidity is 77%) (Xiao et al., 2013). Generally, the seasons can be divided into spring (March to May), summer (June to August), autumn (September to November), and winter (December to February). Rainfall events mainly happened in wet season (May to October). The annual rainfall amount is 900–1,500 mm.

As a heavily contaminated karst city in southwest China (Liu et al., 2017; Xiao et al., 2013), more than 4.5 million inhabitants are living here, presenting a high population density (550 people per km^2^ in 2013). Most of the population is concentrated in the urban area, making the population density up to 800 people per km^2^ and the high degree of urbanization (Figs. 1B and 1C, Fig. S1). The large population results in the emission of atmospheric pollutants, e.g., NO_x_ derived from high level of traffic (more than 680,000 vehicles in Guiyang city). Since 2003, the air SO_2_ concentration in Guiyang has shown a significant decreasing trend, while the air NO_2_ concentration increased gradually (Fig. S2A) (Zhang & Ma, 2016). The annual mean concentrations of air SO_2_ and NO_2_ were 0.031 and 0.033 mg m^−3^ in 2013, and 0.024 and 0.031 mg m^−3^ in 2014 (Yang, Ge & Wu, 2018; Zhang & Ma, 2016). The annual total SO_2_ emission and total NO_x_ emission were about 10.6 × 10^4^ t and 5.7 × 10^4^ t in the study period. The land cover mainly includes water area, urban area, grass land, bare land, farmland, forest, and shrub in the study region (Fig. 1C).

The rainwater samples were collected at the Institute of Geochemistry, CAS, on the central urban built-up area of Guiyang city (26.34 N, 106.43 E, Fig. 1C). This site could well reflect the anthropogenic influence on rainwater in Guiyang city because it is located in the center of the urban built-up area with the highest population density. The polyethylene sampler (set at the place of 15 m over surface) was applied manually to collect rainwater. The polyethylene lid was applied to escape the deposition of atmospheric dust in without-rain days. The sampler was completely cleaned via the deionized-water after each sampling event. The filtration was concluded for each sample by Millipore membrane filters (0.22 μm). The samples after filtration were saved in pre-cleaned polyethylene bottles and kept refrigerated (4 °C). The rainwater samples obtained in each month were proportionally mixed according to the precipitation amount of each precipitation event, and therefore formed a monthly mixed sample. The measured results of mixed sample can denote the monthly weighted-mean values (Jia & Chen, 2010). Ultimately, 26 monthly mixed samples were obtained from May 2012 to June 2014. It is noteworthy that the relatively low rainfall amount was observed in 2013 for Guiyang city with almost no rainfall in July (the dotted box in Fig. S2B). That is, the total rainfall amount in 2013 was 888 mm (only ~70% of the previous annual rainfall), which was also far lower than that in 2012 (1,226 mm) and 2014 (1,562 mm). More details about the daily distribution of rainfall amount can be found in Fig. S3.

### Measurement of rainwater chemicals

Twenty-six samples were divided into two parts and stored in pre-cleaned polyethylene bottles to measure anion and cation concentrations (HNO_3_ acidified, pH < 2). The ion chromatography (IC, DX-120; Dionex Corporation, Sunnyvale, CA, USA) was used to determine the concentrations of major anions, such as NO_3_^−^, SO_4_^2−^. Cl^−^, and F^−^ with the limit of detection (LOD) of 0.06, 0.10, 0.04 and 0.03 mg L^−1^. The ICP-AES (IRIS Intrepid-II; Thermo Scientific, Waltham, MA, USA) was applied for detecting the cations concentrations (Na^+^, Ca^2+^, K^+^, and Mg^2+^) with the LOD of 0.03, 0.04, 0.01 and 0.01 mg L^−1^.

The procedural blanks, standard reference materials (SRM, GBW08606, National Research Center for Certified Reference Materials, China), and replicates were measured together with all samples to ensure quality control. In brief, the satisfactory repeatability and accuracy for all ions (<5%) were observed in replicate samples and SRM. Moreover, all the measured ions in procedural blank were under the LOD or smaller than 5% of corresponding ions in rainwater samples, implying a trustworthy detected procedure.

### Calculation and correlation analysis

To calculate the volume-weighted mean (VWM) concentrations of rainwater ions in the whole study period (May 2012 to June 2014), the equation below is applied (Zhou et al., 2019):

(1) $$C=\frac{\sum C_{i}P_{i}}{\sum P_{i}}$$

where C, C_i_, and P_i_ are the VWM concentration of rainwater ions in the whole study period, the ion concentration of each monthly mixed sample, and the monthly rainfall amount, respectively.

For the statistical analyses of rainwater chemical data, the normal distribution of these data is concluded by the Kolmogorov–Smirnov test (K–S test, a non-parametric test usually used to analyze sample data set in environmental research), and Pearson’s correlation analyses is therefore applied for potential ion source identification. The Pearson’s correlation coefficient is performed using SPSS 21.0 (IBM, Armonk, NY, USA). Furthermore, the representative air contaminants (PM_10_, NO_2_ and SO_2_) are also involved in correlation analysis to clarify the relationship between these species and rainwater ions.

## Results

**Figure 2:**
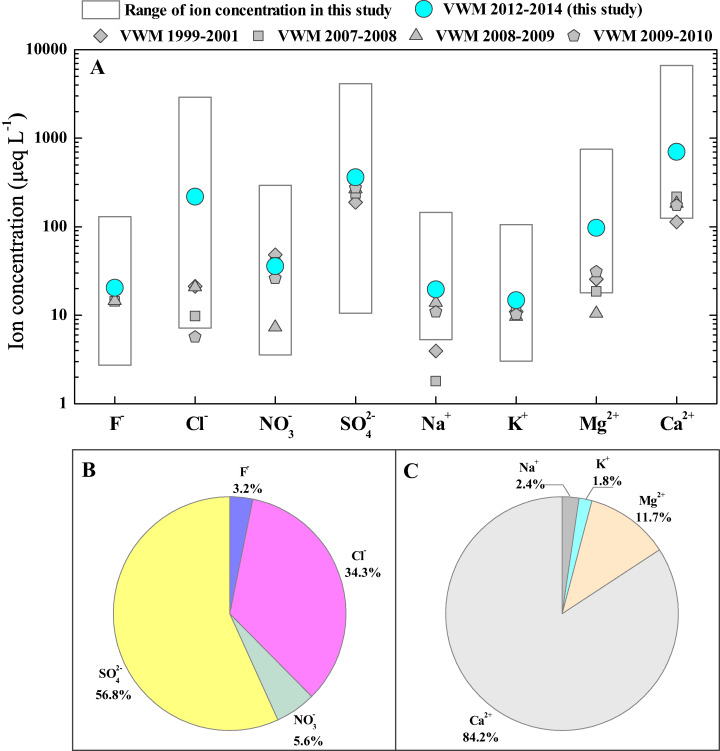
Ion compositions of rainwater in Guiyang city. (A) Overview of rainwater ion contents (μeq L^−1^) of in Guiyang city, data from previous studies was also included (Han & Liu, 2006; Han et al., 2019; Han, Wu & Tang, 2011; Xiao et al., 2013); (B) Rainwater anion composition percentages; (C) Rainwater cation composition percentages.

The statistical results of rainwater ion concentrations (monthly ranges and annual VWM values) are summarized and plotted in Fig. 2A. The significant variations in each ion concentration are observed in Guiyang city. That is, all the concentrations of rainwater ions presented a large range, e.g. the Ca^2+^ concentration ranged from 125 to 6,652 μeq L^−1^ and the concentration of SO_4_^2−^ fluctuated from 11 to 4,127 μeq L^−1^ (Fig. 2A). Thus, the annual VWM concentrations of each ion are more suitable for comparison. The rainwater ion concentrations in this study followed the sequences based on VWM values: Ca^2+^ (700.1 μeq L^−1^) > Mg^2+^ (97.0 μeq L^−1^) > Na^+^ (19.6 μeq L^−1^) > K^+^ (14.8 μeq L^−1^) and SO_4_^2−^ (361.4 μeq L^−1^) > Cl^−^ (218.3 μeq L^−1^) > NO_3_^−^ (35.9 μeq L^−1^) > F^−^ (20.4 μeq L^−1^) (Fig. 2A).

Figures 2B and C presented the percentages of each ion, which indicates that SO_4_^2−^, Cl^−^, Ca^2+^ and Mg^2+^ are the dominant chemical compositions of rainwater in Guiyang city. SO_4_^2−^ and Cl^−^ account for 56.8% and 34.3% of total measured anions, which are the most and second richest anion (Fig. 2B). The SO_4_^2−^ showed the highest percentage, suggesting the potential effect of strong anthropogenic activities (Keresztesi et al., 2019; Zhou et al., 2019). In Fig. 2C, Ca^2+^, Mg^2+^, Na^+^ and K^+^ account for 84.2%, 11.7%, 2.4% and 1.8% of the total detected cations. Thus, Ca^2+^ is the absolute leading cation and Mg^2+^ is also a more important contributor to cations relative to Na^+^ and K^+^. Both geology and urbanization are responsible for the cation compositions. The wide-distributed carbonate in karst region is an important potential source of Ca-enriched dust, which can be washed down by rainfall and further be the significant source of rainwater Ca^2+^ (Han et al., 2019). Additionally, vast Ca-contained pollutants were continually discharge into the atmosphere due the accelerated development of urbanization (construction industry and other human activities). The aqueous calcium hydroxide was generally applied to remove the sulfur dioxide emitted from coal-fired industries (wet desulfurization) (Garea et al., 1996; Wang et al., 2017a), which is another potential source of atmospheric Ca, in particular, since the effective implementation of environmental protection policies, such as National Acid Rain and SO_2_ Pollution Prevention Plan (Wen et al., 2020; Yu et al., 2019). In summary, Ca^2+^ and Mg^2+^ concentrations make up 95.9% of cations, and SO_4_^2−^ and Cl^−^ make up 91.1% of anions.

## Discussion

### Comparison between different zones

We listed the rainwater ion concentration in Guiyang city, together with historical data in Guiyang city, and those published data in various eco-environmental systems (Table 1). In historical comparison, all ion concentrations in the study period (May 2012 to June 2014)were higher than those historical data, with the exception of NO_3_^−^ that slightly lower than 2007 (Fig. 2A and Table 1) (Han & Liu, 2006; Han et al., 2019; Han, Wu & Tang, 2011; Xiao et al., 2013). In particular, the Cl^−^ concentration of this study showed a level comparable to that of oceanic rainwater (Table 1) (Xiao et al., 2016), which reflects that the Cl^−^ was underwent the very strong process of enrichment and concentration (indicates potential anthropogenic input) during the transportation of atmospheric clouds (Jain, Madhavan & Ratnam, 2019), while the HCl-released reaction caused Cl^-^ depletion was negligible. Overall, rainwater ion concentrations in Guiyang city were on the rise. For the horizontal comparison, the concentrations of most ions (Cl^−^, F^−^, SO_4_^2−^, Na^+^, Ca^2+^ and Mg^2+^) of rainwater in this study is higher than that in other karst areas (Table 1), including karst agricultural area (Puding), karst forest area (Maolan), Bohemian karst area (Prague) (Špičková et al., 2008; Zeng et al., 2020). Only the K^+^ and NO_3_^−^ concentrations of rainwater in Guiyang city are lower than those of rainwater in Bohemian karst area (Špičková et al., 2008). This reveals the impact of the different intension of local human emission (e.g., transportation emissions, fossil fuel burning, agricultural production) on rainwater ionic species under various karst environmental types. Moreover, it is noteworthy that the concentrations of SO_4_^2−^ and NO_3_^−^ (typical human-derived ions) in Guiyang city are close to that in inland megacity (Beijing) (Xu et al., 2015), and the concentrations of Ca^2+^ is even higher than that of desert environment (Alxa) (Rao et al., 2017).

**Table 1:** Comparison of ions concentration (in μeq L^−1^) of rainwater at Guiyang city and other published data.

Site	F^−^	Cl^−^	NO_3_^−^	SO_4_^2−^	Na^+^	K^+^	Mg^2+^	Ca^2+^	Rainwater type
Guiyang (this study)	20.4	218.3	35.9	361.4	19.6	14.8	97.0	700.1	Karst city
Guiyang 1999–2001	—	21.2	48.2	188.0	4.0	11.0	25.5	113.2	Karst city
Guiyang 2007	14.3	9.8	39.6	237.8	1.8	11.1	18.6	217.6	Karst city
Guiyang 2008	14.5	20.7	7.3	265.6	13.9	9.6	10.5	182.9	Karst city
Guiyang 2009	19.9	5.7	26.1	274.6	10.9	10.2	62.2	349.4	Karst city
Puding	2.1	7.3	29.6	130.1	18.8	6.1	16.3	119.6	Karst agriculture
Maolan	0.9	5.1	3.0	40.4	2.4	3.5	3.0	20.8	Karst forest
Prague	2.7	18.3	72.9	129.0	8.0	23.4	10.3	319.9	Bohemian karst
Beijing	12.0	50.9	42.6	357.0	21.5	9.2	53.3	273.0	Inland megacity
Shenzhen	—	45.9	23.7	59.3	36.4	2.0	11.8	18.1	Coastal megacity
Alxa	—	202.8	69.7	471.4	232.5	34.1	72.1	663.0	Desert
Eastern Tien Shan	—	16.5	9.6	53.0	19.0	4.0	18.1	174.2	Mountain area
Yongxing island	—	214.4	8.9	38.0	209.7	5.8	45.6	128.8	Oceanic island

**Note:**

“—” denotes no data; data sources: Guiyang 1999–2001(Han & Liu, 2006), Guiyang 2007 (Han, Wu & Tang, 2011), Guiyang 2008 (Xiao et al., 2013), Guiyang 2009 (Han et al., 2019), Puding (Zeng et al., 2020), Maolan (Zeng et al., 2020), Prague (Špičková et al., 2008), Beijing (Xu et al., 2015), Shenzhen (Zhou et al., 2019), Alxa (Rao et al., 2017), Eastern Tien Shan (Zhao et al., 2008), Yongxing island (Xiao et al., 2016).

### Temporal variation, influencing causes and neutralization coefficients of ion compositions

**Figure 3:**
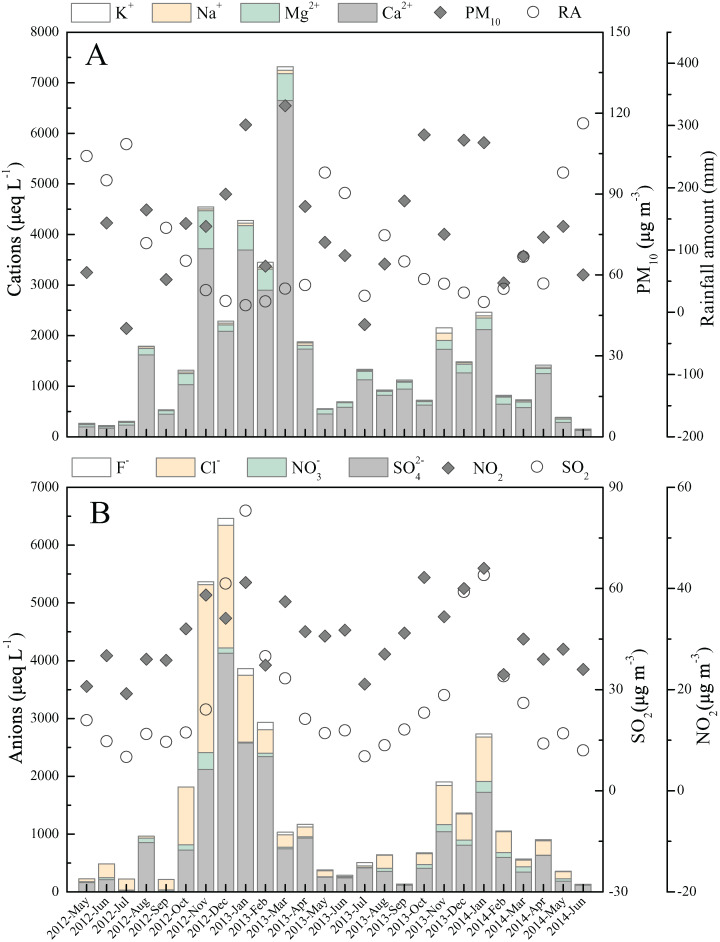
Monthly variations in ion concentrations and other parameters. (A) Cations concentration, PM_10_, rainfall amount (RA); (B) anions concentration and atmospheric SO_2_ and NO_2_ content. The PM_10_ and air SO_2_ and NO_2_ data are from literature (Yang, Ge & Wu, 2018; Zhang & Ma, 2016)

The highest total ion concentrations were found in cooler months (October to March), while the warmer months (April to September) were accompanied by the lowest concentrations (Fig. 3). All ions displayed the parallel temporal change trends, which can be interpreted by two factors. First, the temporal change of potential substance origin is the significant factor of rainwater ions concentrations variation, such as the change of air particulate matter (high content of Ca^2+^) and gaseous air pollutants (Jain, Madhavan & Ratnam, 2019). This can be further supported by the observed monthly variations in air PM_10_, SO_2_, and NO_2_ content, that is, the high/low concentrations of rainwater Ca^2+^ was accompanied by high/low air PM_10_, SO_2_ and NO_2_ content (Figs. 3A and 3B). These related parameters show significant positive correlation. The correlation coefficients (*p* < 0.05) are 0.58, 0.74 and 0.51 between Ca^2+^ and air PM_10_, SO_4_^2−^ and air SO_2_, and NO_3_^−^ and air NO_2_, respectively. Second, the relative frequent rainfall event in wet season (warmer months) can effectively wash down the various atmospheric components, and further result in the air contaminants could not have a long retention time compared to dry season (warmer months) (Xiao & Liu, 2002), while more air components are scoured by rainwater in dry season with little rainfall (Szép et al., 2019).

**Figure 4:**
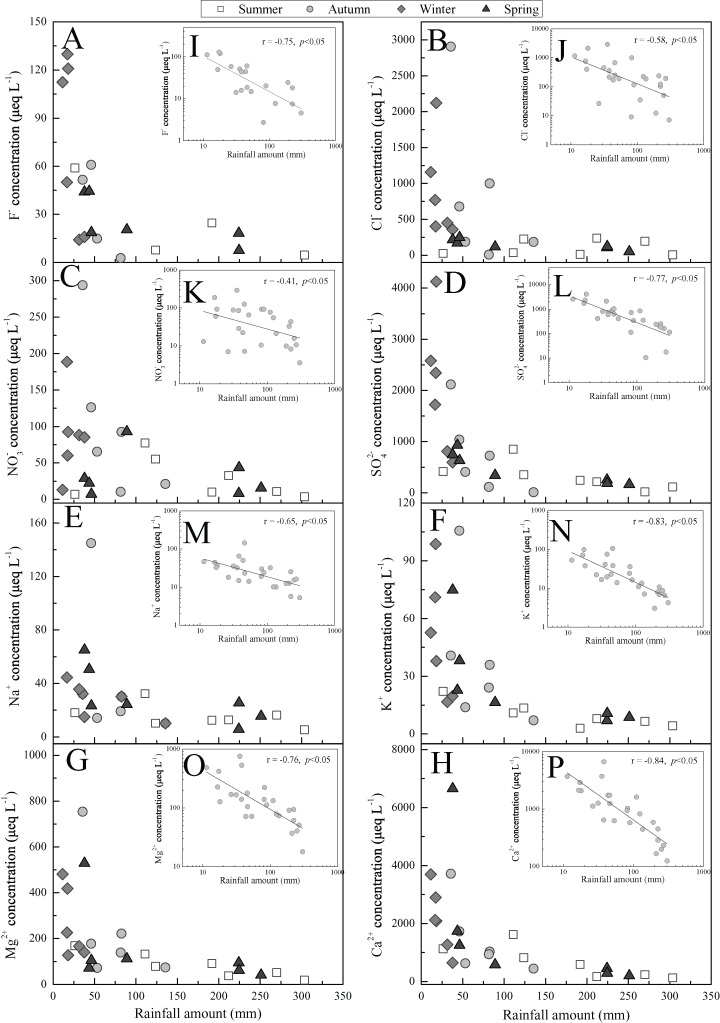
Relationships between ions concentrations and rainfall amount in Guiyang city from 2012-Summer to 2014-Spring. A–H represent the relationships between rainfall amount and F^−^, Cl^−^, NO_3_^−^, SO_4_^2-^, Na^+^, K^+^, Mg^2+^, and Ca^2+^; I–P are the corresponding logarithmic ion content vs. logarithmic rainfall amount.

Overall, the ions concentrations presented a converse temporal trend to the precipitation amount from the monthly scale (Fig. 3). This revealed that precipitation amount is the important factor impacting rainwater ionic species, which is in agreement to the previous findings (Keresztesi et al., 2020b; Szép et al., 2018). The more clear relationship between rainfall amount and ions concentrations were illuminated in Figs. 4A–4H. The further correlation analysis of ions concentrations and precipitation amount after logarithmic computation (Figs. 4I–4P) revealed that ions concentrations in Guiyang city are observably impacted by the dilution effect (or scouring effect) under large rainfall amount (e.g., r = −0.84 for Ca^2+^ and r = −0.77 for SO_4_^2−^, *p* < 0.05), which is commonly found in other areas (Silva, Gonçalves & Freitas, 2009; Szép et al., 2018). In this scouring process, the air components, such as sulfur/nitrogen oxides, Mg and Ca-mineral, and particulate nitrate (e.g., nitrated phenols (Li et al., 2020b)) are powerfully scoured down (below-cloud processes) during the early rain-stage, causing the high ionic concentration observed under the low precipitation amount condition (Jia & Chen, 2010). In contrast, without the continuous supplements of gaseous air contaminants or suspended matters, the ionic concentration of the late rain-stage (prolonged precipitation event) is decreased progressively and stayed at a low content level (Figs. 4A–4H) (Gonçalves et al., 2002). It is noteworthy that the critical point to distinguish between early and late rain-stage is ~100 mm for all ions from Figs. 4A–4H. This indicates that the various species in the atmosphere are almost washed down completely within the precipitation of 100 mm, and the similar critical points (different rainfall values) were observed in previous studies (Silva, Gonçalves & Freitas, 2009; Xiao et al., 2013; Zeng et al., 2020). Conversely, if the precipitation amount exceeds 100 mm, the rainwater ion concentrations decreased insignificantly with the increase of rainfall amount and even stayed at a basically constant and very low concentration level (Figs. 4A–4H). At this time, the rainwater ionic contents primarily reveal in-cloud processes, that is, the ionic concentration of rainwater is similar to the cloud-water (gas/aerosols-dissolved cloud drops) (Rao et al., 2017; Silva, Gonçalves & Freitas, 2009). Under such condition (precipitation >100 mm), the scouring effect is negligible, while the long-range transportation is regarded as the most critical reason influencing rainwater ionic compositions (Gonçalves et al., 2002).

To gain more information regarding to the neutralization coefficients of different alkaline ions in rainwater, the VWM concentrations-based neutralization factors (NFs) were calculated according to the equation: NF of X = [X]/[NO_3_^−^ + SO_4_^2−^], where X= Na^+^, K^+^, Mg^2+^, Ca^2+^ (Xiao et al., 2013). The NF values of Na^+^, K^+^, Mg^2+^ and Ca^2+^ in rainwater at Guiyang city were 0.05, 0.04, 0.24 and 1.76, respectively. These results suggest that Ca^2+^ is the dominant neutralization alkaline ion in the rainwater, following by the ions of Mg^2+^, Na^+^, and K^+^, which also indicate the significant potential influence of Ca-enriched substances derived from the construction industry and windblown dust. Furthermore, the enrichment factors (EF) of ions in rainwater relative to the corresponding ions in seawater/crust were calculated as: EF_seawater_=[X/Na^+^]_rainwater_/[X/Na^+^]_seawater_; EF_crust_ =[X/Ca^2+^]_rainwater_/[X/Ca^2+^]_crust_, where the ion equivalent ratios of [X/Na^+^]_seawater_ and [X/Ca^2+^]_crust_ were followed the previous work (Berner & Berner, 1987; Rudnick & Gao, 2004). The EF_seawater_ of K^+^, Mg^2+^, SO_4_^2−^ and Ca^2+^ was calculated as 35, 22, 152 and 814, while the EF_crust_ of K^+^, Mg^2+^ and Na^+^ were 0.05, 0.14 and 0.03. These results significantly suggest that most of the rainwater ions were concentrated relative to the seawater, but depleted relative to the earth’s crust.

### Source of major ions

**Figure 5:**
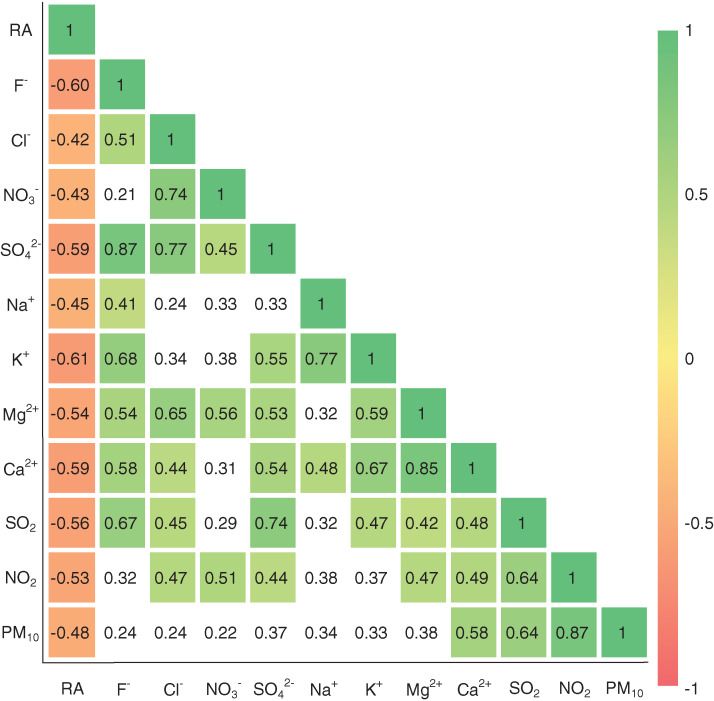
Pearson correlation coefficients between major ions in rainwater and other parameters in Guiyang city. RA, rainfall amount; white cells denote statistically non-significant correlations (*p* > 0.05), while other cells denote the significance level of *p* < 0.05.

Since the alike physico-chemical natures and potential co-sources of some atmospheric components (e.g., SO_x_ and NO_x_) (Vet et al., 2014), the correlation coefficient is a frequently-used tool for exploring the common origins of rainwater chemical components (Xing et al., 2019), which is applied in this study. As shown in Fig. 5, the significant negative correlations are presented between precipitation amount and all ions and air pollutants (*p* < 0.05), which further confirms the important effect of precipitation amount on the variations in ionic concentrations. However, obvious positive correlations are observed between most of ion components, such as r = 0.85 for Ca^2+^ and Mg^2+^, r = 0.87 for SO_4_^2−^ and F^−^, r = 0.77 for SO_4_^2−^ and Cl^−^, and r = 0.45 for SO_4_^2−^ and NO_3_^−^ (Fig. 5), it is thereby difficult to obtain further origin information of rainwater ion by correlation analysis.

**Figure 6:**
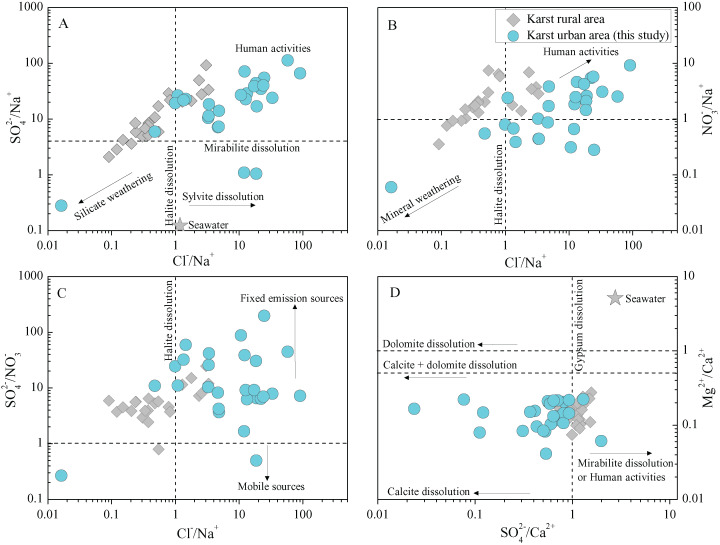
The relationship between the ratios of typical ions (equivalent ratio) in karst urban and rural rainwater. (A) SO_4_^2−^/Na^+^ vs. Cl^−^/Na^+^, (B) NO_3_^−^/Na^+^ vs. Cl^−^/Na^+^, (C) SO_4_^2−^/NO_3_^−^ vs. Cl^−^/Na^+^, and (D) Mg^2+^/Ca^2+^ vs. SO_4_^2−^/Ca^2+^ ratio. Data sources for karst rural rainwater chemistry and other reference values are from (Berner & Berner, 1987; Zeng et al., 2020).

To identify further the associations of various ions of rainwater and the potential origins, the representative ionic ratios (in equivalent concentration, e.g., Cl^−^/Na^+^, SO_4_^2−^/NO_3_^−^ and Mg^2+^/Ca^2+^) are presented in Fig. 6A. The low Cl^−^/Na^+^ ratios (compared with seawater) are only found in few samples, while most samples present a higher Cl^−^/Na^+^ ratio relative to seawater (Berner & Berner, 1987). In total, the rainwater Cl^−^/Na^+^ ratios in this study (karst urban area) are also much greater than that in karst agricultural regions (Zeng et al., 2020). In combined with the high Cl^−^ concentrations (comparable to the Cl^−^ in marine rainwater) and relative low Na^+^ concentrations (Table 1), here we conclude that the sea salt input Cl^−^ was intensively enriched in the transported processes of atmospheric clouds (Jain, Madhavan & Ratnam, 2019; Xiao et al., 2013), while the Na^+^ may be reduced in some degree due the input of other cations. That is, there are other potential sources of Cl^−^ besides marine sources. The relationship between Cl^−^ and the typical anthropogenic inputs SO_4_^2−^ (r = 0.77 for SO_4_^2−^ and Cl^−^) further support this. Previous work has shown that the chlorinated chemical plants emissions and combustion of chlorinated organics in some coal combustion industries are the important anthropogenic sources of rainwater Cl^−^ (Facchini Cerqueira et al., 2014; Keresztesi et al., 2019; Wu et al., 2012). Moreover, the facilities using large amounts of disinfectants (Cl-contained) is also another potential source of rainwater Cl^−^, such as the hospitals near the sampling site (Guizhou Electric Power Workers Hospital, and Guiyang First People’s Hospital) and other place (e.g., swimming pool). Thus, here we attributed the high contents of rainwater Cl^−^ were caused by the potential coal combustion industries, disinfectants-used facilities and the Cl^−^ enrichment in long-distance transportation. Furthermore, most rainwater samples present high SO_4_^2−^/Na^+^ and NO_3_^−^/Na^+^ ratios (Figs. 6A and 6B), indicating the significant impact of anthropogenic input (human activities), that is, these anions (SO_4_^2−^ and NO_3_^−^) are mainly derived from anthropogenic emissions (Keresztesi et al., 2019; Xu et al., 2015). Especially, SO_4_^2−^/NO_3_^−^ ratio exceed 1 is observed in almost all samples, even up to 198 (Fig. 6C), further reflecting the leading role of fixed contamination emission origins, e.g., transportation/diffusion of the coal-burning emissions (Li et al., 2020a; Naimabadi et al., 2018), whereas the mobile sources contribution (mainly vehicle emissions) and its long-range transport/diffusion are relative muted (Rao et al., 2017). In Fig. 6D, all the sample symbols are plotted between the lines of (calcite + dolomite dissolution) and (calcite dissolution) due to the small Mg^2+^/Ca^2+^ ratios (0.04–0.22), implying the primarily influence of calcite dissolution of atmospheric dust derived from different carbonate weathering processes on Ca^2+^ and Mg^2+^ in rainwater (Keresztesi et al., 2020a; Lü, Han & Wu, 2017). On the contrary, the sea salt contribution to the rainwater cations is very limited (based on the comparison with seawater cations ratio in Fig. 6D). In addition, although F^−^ and K^+^ related ionic ratios are not shown in Fig. 6, the previous work has indicated that the biomass burning (e.g., straw combustion) and soil dust are the main sources of K^+^ and the anthropogenic emissions can be a interpretation for the higher F^−^ concentration in rainwater (Khare et al., 2004; Zhang et al., 2019). Moreover, although the rainwater NH_4_^+^ was not measured in the present study due to the inherent difficulty of the instrument (ICP-AES) for cations measurement, the previous rainwater nitrogen isotope-based studies have concluded that the rainwater NH_4_^+^ in Guiyang mainly from animal/human wastes (~22%), fertilizers (~22%), and agricultural biomass burning (~17%)(Liu et al., 2017; Xiao et al., 2012). This is highly in agreement with the characteristics (full of agricultural activities around the city and concentrated population in urban area) in such a developing city in southwest of China.

### Deposition flux and potential environmental effects

Based on the ionic concentrations and the corresponding rainfall amount in each month during study period, the rainwater ions deposition fluxes are estimated (Fig. S4). There are fairly large ion deposition fluxes in such a karst city intensively impacted by human activities, with the range of 0.5 to 3.5 keq ha^−1^, which is also a very high level over China (0.1–2.5 keq ha^−1^) (Rao et al., 2017; Yang et al., 2012; Zhao et al., 2008; Zhou et al., 2019). As expected, the lowest monthly wet deposition flux is observed in July 2013 (Fig. S4) due to the extremely low rainfall amount in this month (26.4 mm) compared to the rainfall amount in other years (about 270–420 mm, Fig. S2B). These results suggest that both the sources contribution and the climatic conditions (e.g., rainfall amount) are responsible for the total ions wet deposition fluxes.

Moreover, the combination of protons in the aquatic ecosystems and nitrate and sulfate derived from wet deposition actuated water acidification in the hydrosphere have been a focus of environmental researches (Chen et al., 2019; Qiao et al., 2018), the wet deposition levels of sulfate and nitrate can therefore reflect the potential environmental effects of rainwater chemistry to some extent (Pan et al., 2013). This phenomenon significantly impacted the carbonate weathering in karst areas further influenced the global climate change (Li et al., 2008; Liu & Han, 2020; Liu & Han, 2021; Raymond et al., 2013; Wang et al., 2019). The previous studies on the aquatic ecosystem in Guiyang city also showed the enhanced carbonate rock weathering processes involved with exogenous sulfuric and nitric acid (Lang et al., 2006; Zhong et al., 2018). The monthly wet acid (nitrate and sulfate) deposition at Guiyang city ranged between 0.04 to 1.03 keq ha^-1^ (Fig. S5). The evaluated yearly wet acid deposition is 4.27 keq ha^−1^ year^−1^ via the sum of each month’s fluxes in 2013. This annual deposition flux is twice as much as in karst rural areas (Zeng et al., 2020). Different from the Yangtze River Delta (nitrate-dominated wet acid deposition) (Chen et al., 2019), the wet acid deposition in Guiyang city is controlled by the sulfur. The sulfur contributed significantly higher than the nitrate to total acid deposition with the mean monthly contribution rate of 88% (Fig. S5). Thus, the more detailed spatio-temporal monitoring of rainwater chemistry in karst urban area is necessary for understanding the rainfall-actuated acid deposition and its potential effects on hydrosphere, especially sulfate wet deposition (as an agent of weathering).

## Conclusions

In conclusion, 26 monthly mixed rainwater samples collected in a karst city (Guiyang, 2012 to 2014) were investigated to explore the variations, influence factors and origins of ions. The rainwater ions composition varied obviously based on temporal rainwater chemistry investigation. Especially, Ca^2+^ and SO_4_^2−^ concentrations range from 124.5 to 6,652.4 μeq L^−1^ and from 10.5 to 4,127.2 μeq L^−1^, respectively. Ca^2+^, Mg^2+^, SO_4_^2−^ and Cl^−^ are the most dominant ions with obviously monthly variations. Sources variations and precipitation amount are the vital affecting factors of rainwater ions, and the long-distance transportation is also non-negligible factor when precipitation amount exceed 100 mm. Source identification indicated that Mg^2+^, Ca^2+^ and K^+^ are predominantly influenced by the crustal sources (dust), while the anthropogenic emissions are the main origins of F^−^, NO_3_^−^ and SO_4_^2−^. Cl^−^ Enrichment in long-distance transport is the main contributor of Cl^−^. The observed high level of total wet acid deposition implies the requirement of focus on rainfall-related acid deposition, particularly sulfur deposition as which is a potential weathering agent.

## Supplemental Information

10.7717/peerj.11167/supp-1 Supplemental Information 1 The more details of land use of the surrounding of sampling site.

10.7717/peerj.11167/supp-2 Supplemental Information 2 The concentrations of air SO_2_ and NO_2_, and rainfall amount at Guiyang city. (A) The concentration variations of air SO_2_ and NO_2_ at Guiyang city since 2003; and (B) the monthly rainfall amount in 2012, 2013, and 2014.

10.7717/peerj.11167/supp-3 Supplemental Information 3 Daily distribution of rainfall amount in Guiyang city. (A) 2012, (B) 2013, and (C) 2014.

10.7717/peerj.11167/supp-4 Supplemental Information 4 Monthly wet deposition fluxes of ions at the Guiyang city.

10.7717/peerj.11167/supp-5 Supplemental Information 5 Wet acid deposition of nitrate and sulfur in Guiyang city. The percentage in the columns are the proportions of sulfur deposition.

10.7717/peerj.11167/supp-6 Supplemental Information 6 Raw data of measured ion concentrations of rainwater and rainfall amount.

## Additional Information and Declarations

### Data Availability

The following information was supplied regarding data availability: Raw measurements are available in the Supplemental Files.
