# Supplementary figures and images for: Rainwater chemistry observation in a karst city: variations, influence factors, sources and potential environmental effects
Source: PeerJ. 2021 Apr 20;9:e11167. doi: 10.7717/peerj.11167 (PMC8065247; doi:10.7717/peerj.11167)

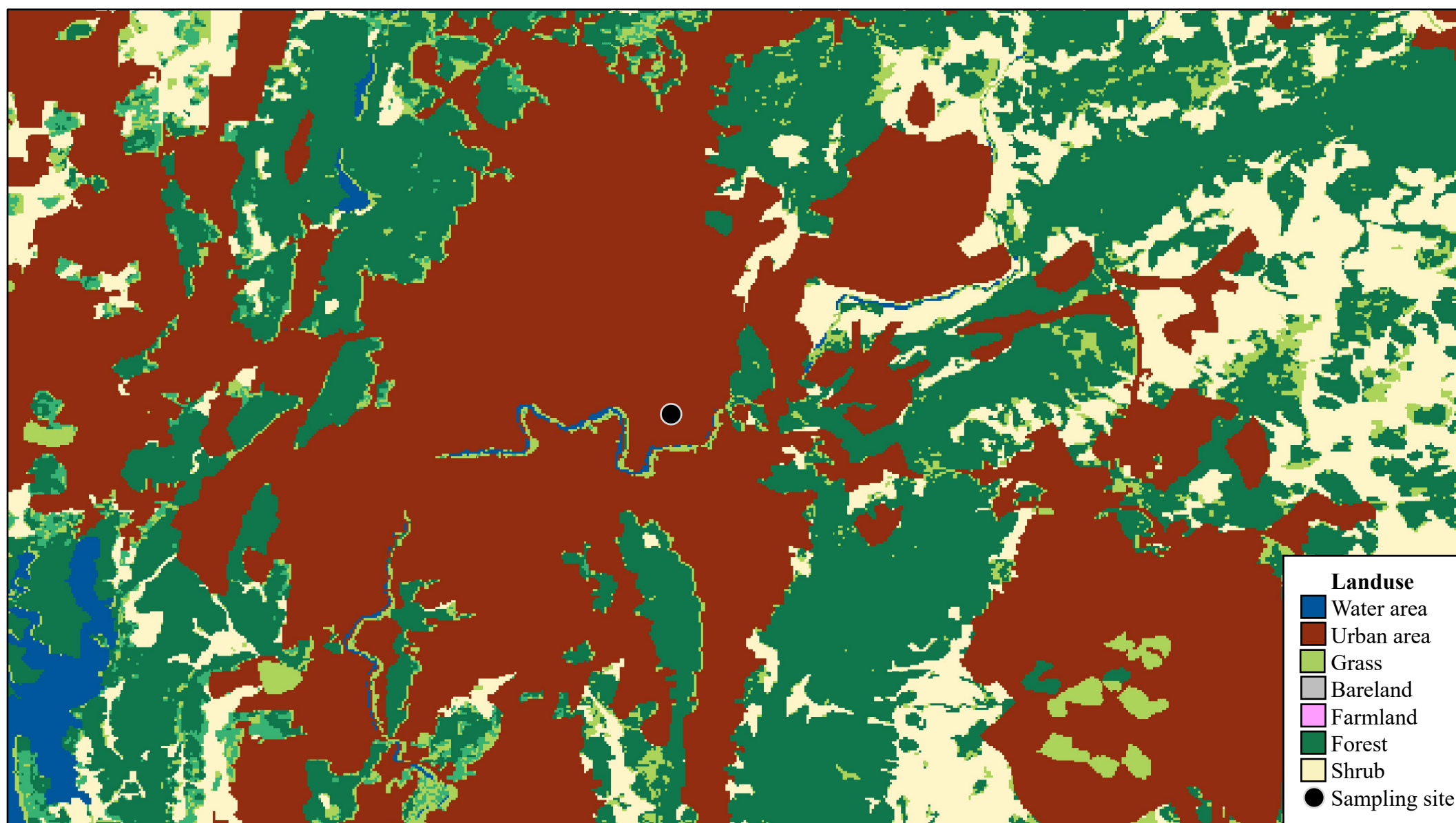

Supplement: Supplemental Information 1 [file peerj-09-11167-s001.pdf]

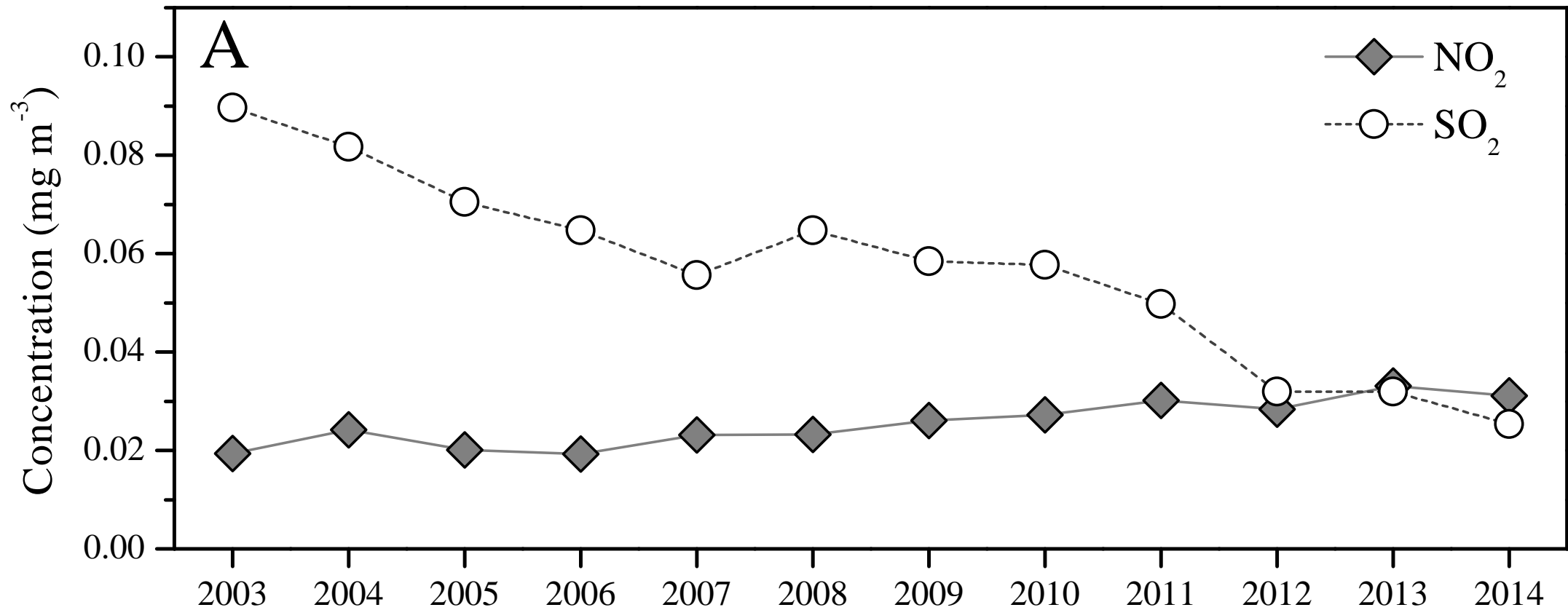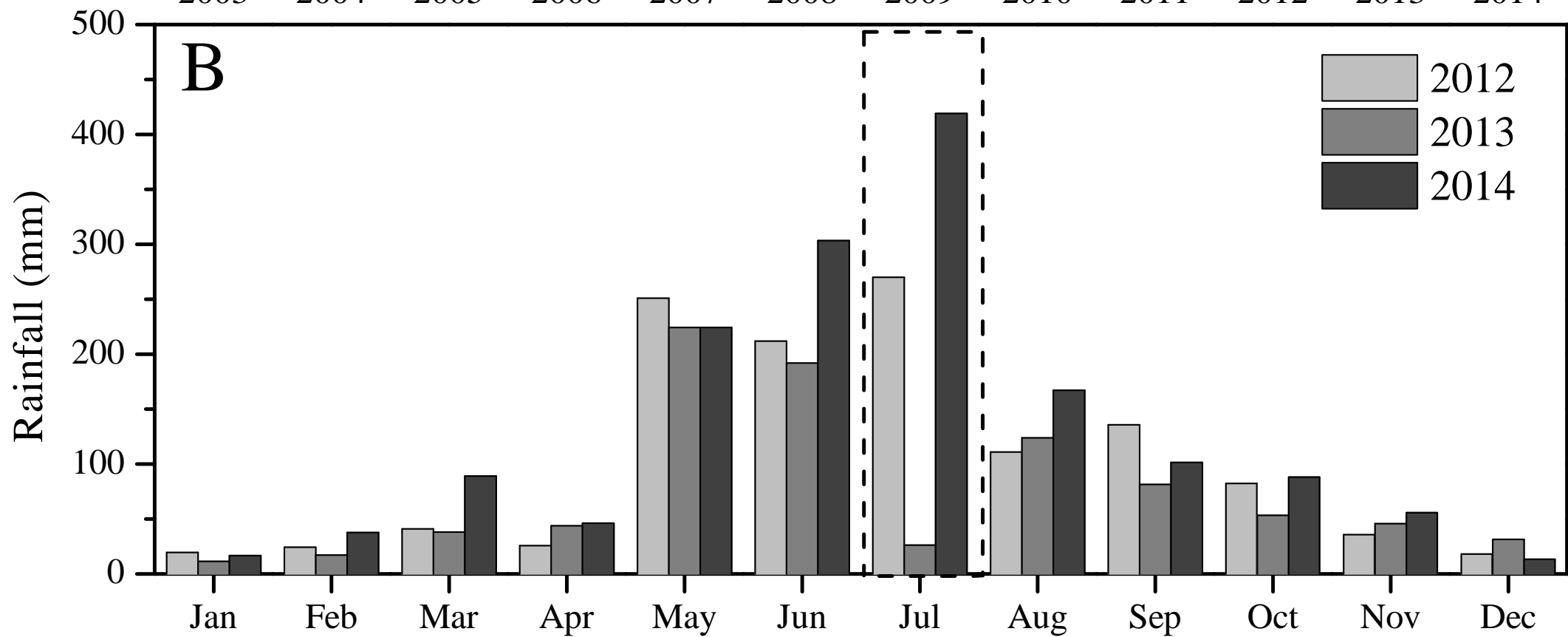

Supplement: Supplemental Information 2 — (A) The concentration variations of air SO2 and NO2 at Guiyang city since 2003; and (B) the monthly rainfall amount in 2012, 2013, and 2014. [file peerj-09-11167-s002.pdf]

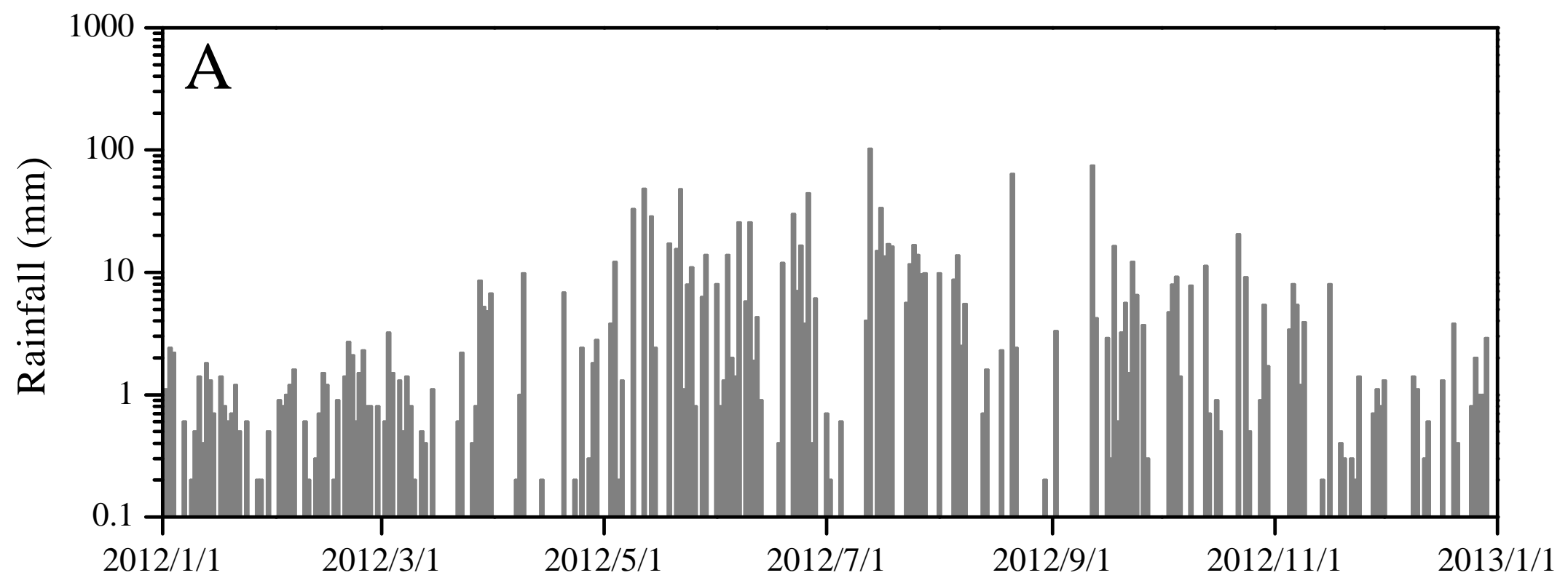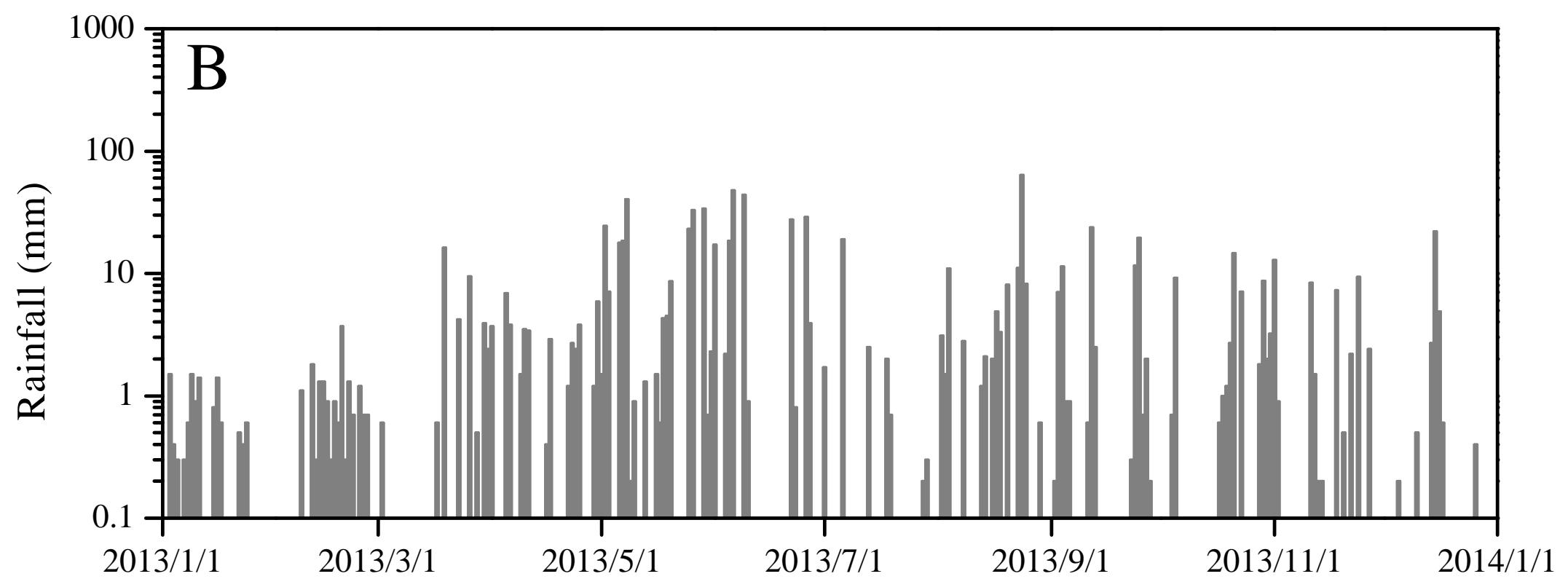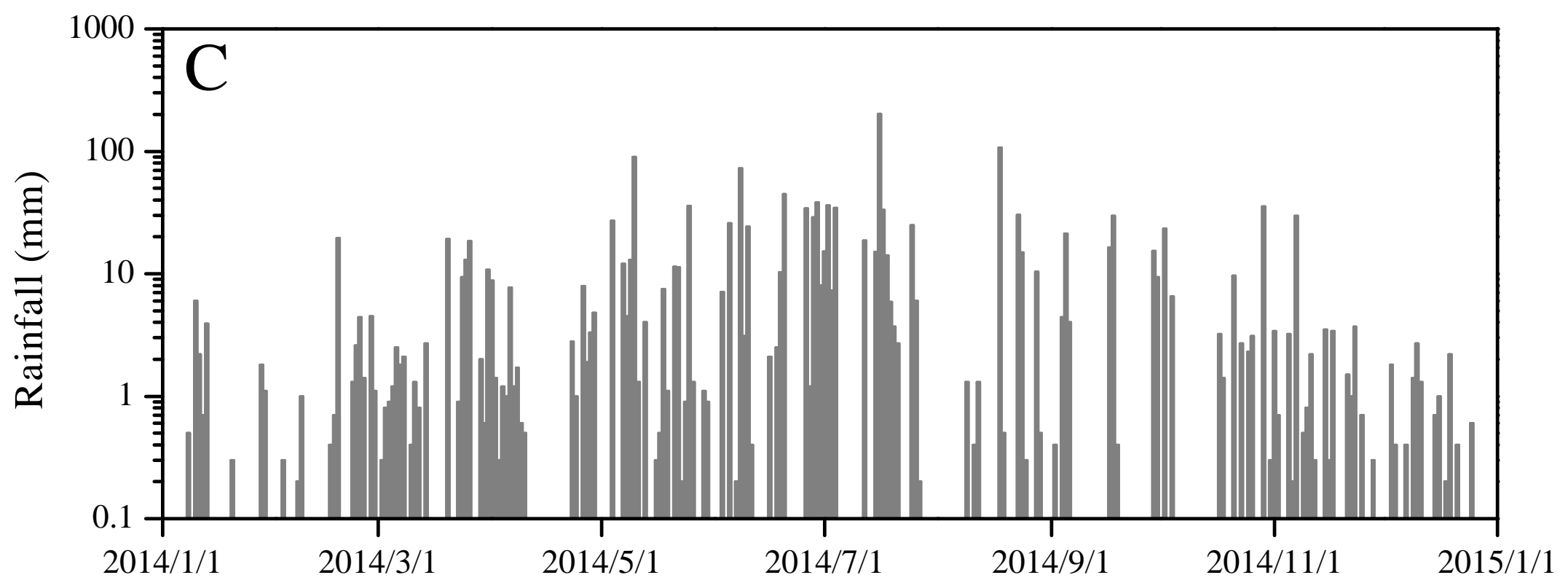

Supplement: Supplemental Information 3 — (A) 2012, (B) 2013, and (C) 2014. [file peerj-09-11167-s003.pdf]

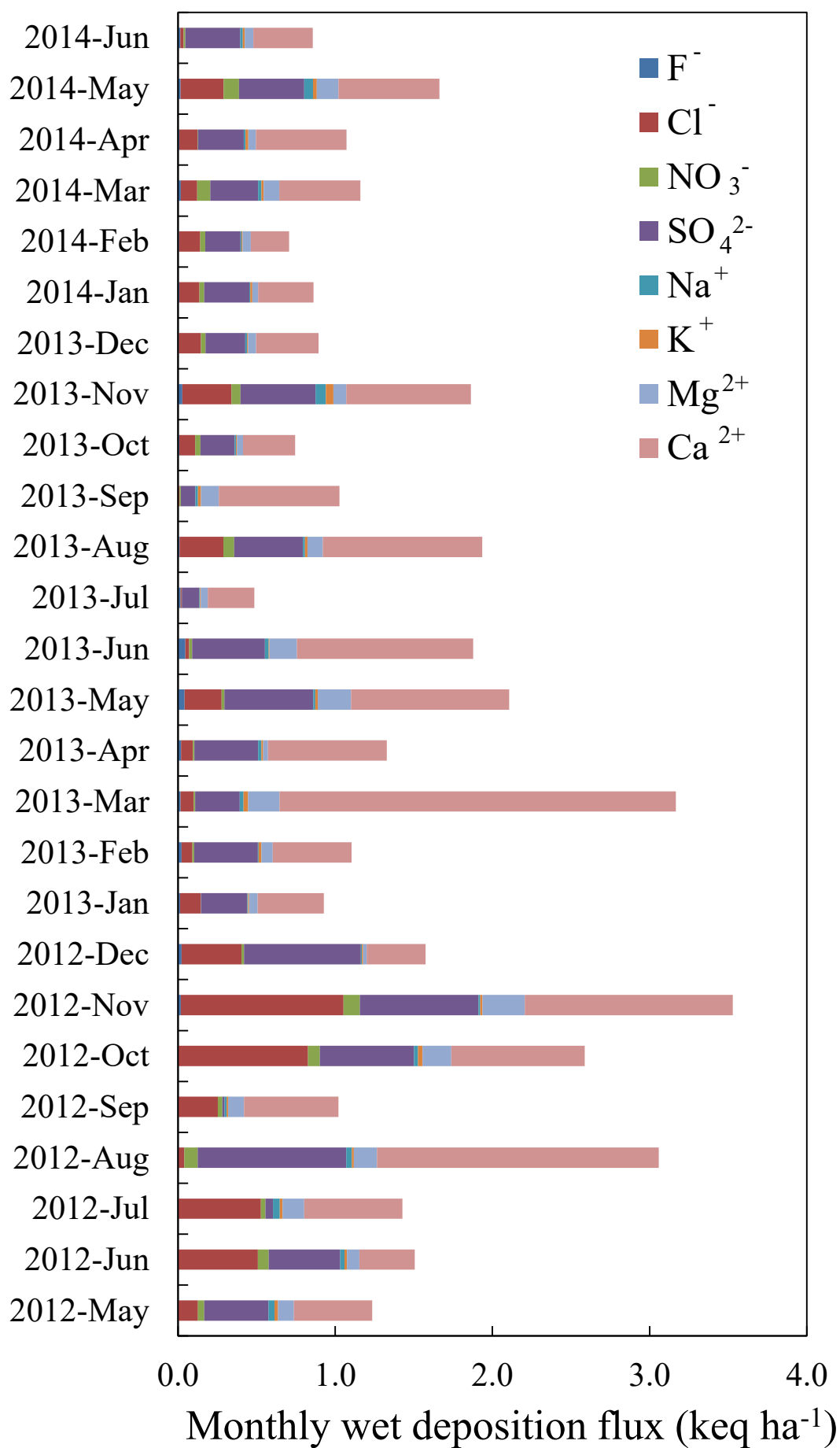

Supplement: Supplemental Information 4 [file peerj-09-11167-s004.pdf]

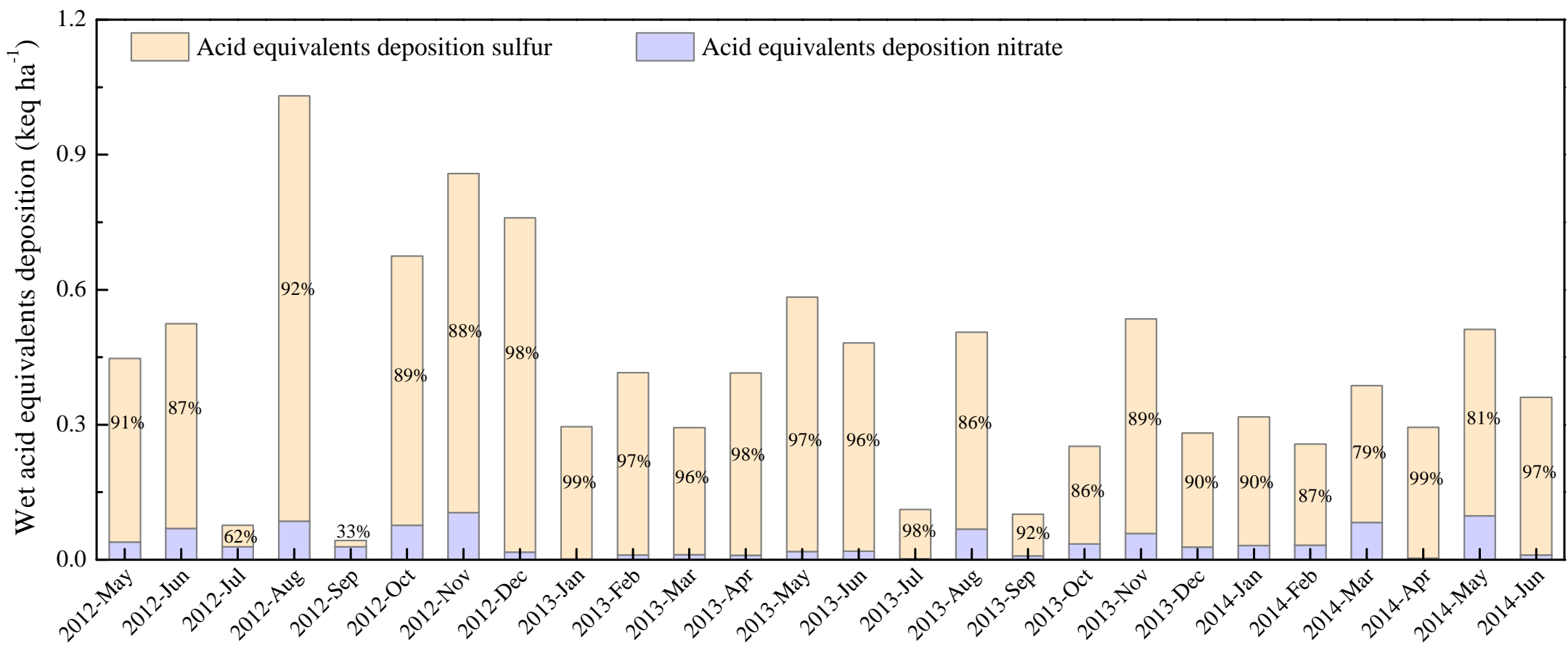

Supplement: Supplemental Information 5 — The percentage in the columns are the proportions of sulfur deposition. [file peerj-09-11167-s005.pdf]
